# Discrimination of boron tolerance in *Pisum sativum* L. genotypes using a rapid, high-throughput hydroponic screen and precociously germinated seed grown under far-red enriched light

**DOI:** 10.1186/s13007-017-0221-3

**Published:** 2017-08-29

**Authors:** Richard G. Bennett, Federico M. Ribalta, Maria Pazos-Navarro, Antonio Leonforte, Janine S. Croser

**Affiliations:** 1 Centre for Plant Genetics and Breeding, The University of Western Australia, 35 Stirling Hwy, Perth, WA 6009 Australia; 2 Nuseed, 5 Ballinger St, Horsham, VIC 3400 Australia

**Keywords:** LED, Boron tolerance, Immature seed, Leaf symptoms, Abiotic screening

## Abstract

**Background:**

Boron (B) tolerance has been identified as a key target for field pea improvement. Screening for B tolerance in the field is problematic due to variability in space and time, and robust B molecular markers are currently unavailable in field pea. There has been recent progress in developing protocols that can accelerate the life cycle of plants to enable rapid generation turnover in single seed descent breeding programs. A robust B screening protocol that can be fully integrated within an accelerated single seed descent system could lead to rapid identification and introgression of B tolerance into field pea genotypes. Integration with an accelerated single seed descent system requires: (1) screening under artificially lit, temperature-controlled conditions; (2) capacity to use immature precociously germinated seed (PGS); (3) recovery of lines without significant time penalty; and (4) good correlation with results from established screening protocols.

**Results:**

We present herein a B toxicity screening system for field pea based on hydroponic growth of PGS in a light and temperature controlled environment that allows recovery of seedlings for rapid seed production. Screening results were compared to traditional methods for B tolerance screening in B-laced soil and with published field tolerance ratings. B tolerance was scored 17 days after sowing using leaf symptoms as a metric. Plants were then transferred to soil with maximum of six days delay in flowering compared to a typical accelerated single seed descent system generation. The use of PGS had minimal impact on B tolerance rankings compared to plants grown from mature seed. The leaf tolerance rankings from hydroponic-grown plants correlated well with those from soil-grown plants, and consistently identified the most tolerant genotypes.

**Conclusions:**

Our 17 day screening protocol represents a major time-saving over previously published B screening protocols for field pea, thereby extending the application of the protocol to traditional single seed descent systems or RIL screening. We anticipate that small modifications to the proposed technique will make it applicable to screen for other individual abiotic stresses, or allow studies of the interactions between B tolerance and stresses such as salinity.

**Electronic supplementary material:**

The online version of this article (doi:10.1186/s13007-017-0221-3) contains supplementary material, which is available to authorized users.

## Background

Field pea (*Pisum sativum* L.) is an important grain and forage crop grown widely in temperate and Mediterranean climates for human and animal consumption [1]. Field pea provides benefits to cereal-based rotations as a break crop for pest and disease management and improves soil fertility from the rhizobial fixation of atmospheric nitrogen [2]. Along with improved yield, nutritional quality, adaptation and reliability, field pea breeders have identified boron (B) tolerance as an important target for improvement [3]. Boron toxicity is caused by high B concentration in soil or irrigation water, and is particularly problematic in medium or heavier textured soil types with moderate alkalinity and low annual rainfall (<500 mm p.a.) where leaching of B from the soil is slow [4–7]. Yield losses due to B toxicity are hard to quantify. While losses in field pea crops have not been measured directly, yield penalties of 10–20% have been estimated in wheat and barley field crops due to B rich soils [4, 8]. Remediation of boron-toxic soils is impractical in most cases so genetic solutions based on improved plant tolerance to B have been investigated for several decades by plant breeders [6].

A system for rapid generation cycling in pulses has recently been developed that can speed up breeding and molecular mapping efforts. Accelerated single seed descent (aSSD) employs techniques to speed flowering and precociously germinate immature seed [9–11]. In field pea, the aSSD system enables the turnover of up to six generations per year across a diverse range of phenologies [11]. The integration of a B tolerance screen within this aSSD system will facilitate the rapid development of novel genotypes with tolerance to B. However, to enable integration into the aSSD platform, a screen needs to fulfil four key requirements: (1) Occur within a controlled environment growth facility to enable rapid, year-round screening; (2) Have the capacity to use immature precociously germinated seed (PGS) as a starting material; (3) Enable recovery of lines without a significant penalty to generation turnover time; (4) Produce a result that correlates well with established screening protocol. To date, there is no reported B screening protocol that can satisfy these requirements.

A recurrent problem in field screening for abiotic stress tolerance is environmental heterogeneity. The severity of climatic, abiotic and biotic stresses such as drought, frost, subsoil constraints or disease can all vary in the field year to year and within a site, confounding screening results [12]. Even under temperature controlled glasshouse conditions, seasonal changes in photoperiod and light intensity lead to changes in photosynthetic load and transpiration, affecting the repeatability and accuracy of abiotic stress screening in traits that are linked to water use, such as B tolerance [13]. Extended photoperiod, far red-enriched light and tailored temperature in a growth chamber are used to reduce generation time and minimise seasonal influences in the aSSD system [9, 11], but there are no reports of B tolerance screening in this type of environment. There is clear value in the development of a controlled environment-based screen to enable rapid, repeatable and uniform discrimination for B tolerance in field pea.

The use of immature PGS is a key element of the aSSD system [9, 11]. However, to our knowledge, no screening method has used immature PGS as a starting material. With the appropriate treatment, immature seeds have the capacity to robustly germinate once the embryo reaches physiological maturity and, in field pea, this occurs at 18 days after anthesis under aSSD growth conditions [11]. Active exclusion mechanisms play a role in B tolerance [14], so the energy available from the germinating seed may affect the ability of seedlings to tolerate B stress during early plant growth. However, seed development after physiological maturity is reached is primarily related to desiccation [15], rather than energy accumulation, and Ribalta et al. [11] saw no developmental penalty in plants grown from immature PGS, once physiological maturity was achieved. Therefore, we expect to observe similar responses to B stress in seedlings grown from immature PGS, compared to mature seed.

A reliable screening system needs to facilitate the rapid assessment of a large number of individuals and be based on accurate and simple selection criteria [12]. To integrate with aSSD it is also essential that screened individuals can be recovered to produce at least one seed for the following generation with minimal time delay. The screen, therefore, needs to employ a reliable, non-destructive tolerance metric and a growth system carried out within a light and temperature controlled environment similar to a regular aSSD generation. Two non-destructive metrics available for B tolerance in field pea are molecular markers and leaf toxicity symptoms. The molecular markers currently available are not yet fully reliable [3, 16], whereas the simplicity and ease of use of leaf toxicity symptoms has led to them being the benchmark metric when screening field peas for B tolerance [3, 13, 16–18]. Regarding an ideal growth system for controlled environments, hydroponic systems allow plants to be grown compactly, which makes them suitable for use in controlled environments and enables throughput of a large number of individuals, while allowing precise and uniform exposure of plants to abiotic stresses [19–21]. Unlike soil-based screening methods, hydroponic systems also allow for plants to be easily removed from an abiotic stress after screening has occurred by either altering the hydroponic solution or by transplanting to a suitable soil medium. We reason that the application of hydroponic growth systems under controlled conditions using leaf symptoms as the B tolerance discrimination metric, and recovery of hydroponically grown plants to pots for seed production should allow efficient integration of a B tolerance screen within aSSD-based genetic improvement programs.

This paper presents a B toxicity screening system for field pea that is based on hydroponic growth of immature PGS in a light and temperature controlled environment and which allows recovery of seedlings for seed production. We compare the results from this novel screening system to the traditional methods of B tolerance screening in B-laced soil and published tolerance ratings for field pea genotypes. Specifically, our hypotheses were: (1) This screening methodology would provide good correlations with traditional metrics and established rankings; (2) Symptoms of B toxicity in plants grown from immature PGS will be similar to those from mature seed; (3) Screening in a controlled environment, followed by recovery of seedlings to soil would allow the screen to be carried out rapidly and without significant delay to the aSSD generation. Aside from providing a protocol for B tolerance screening that can be applied to traditional breeding and selection systems, the integration of a repeatable and consistent screen into the aSSD system should lead to more rapid development of B tolerant field pea cultivars.

## Methods

The research was undertaken within the controlled plant growth facilities at the University of Western Australia, Perth (lat: 31°58′49″; long: 115°49′7″). Field pea genotypes were selected in consultation with the Pulse Breeding Australia (PBA) field pea breeding team to represent a range of B tolerance based on published B tolerance rankings for commercial cultivars that were established using pot trials [22], hereafter ‘DEDJTR data’, and breeders’ advice for pre-release lines (Dr Antonio Leonforte, unpublished data). Through consultation with breeders and pre-breeders (pers. comm. Drs. Matthew Rodda, Timothy Sutton and David Peck) and a preliminary experiment, we developed a hydroponic system for assessing B sensitivity in field pea when grown under controlled environment conditions. This paper reports on the development of the hydroponic system and validation through comparison with conventional B tolerance screening techniques, including biomass production, flowering behaviour and seed yield of plants grown in pots (Additional file 1: Table S1).

Since harvesting of immature seed is a key element of the aSSD system [9, 11], hydroponic experiments were designed to determine if seed maturity had an effect on the response to B. Immature seeds (at the embryo physiological maturity stage, 18 DAA) were produced for all the genotypes (Additional file 1: Table S1) and subjected to treatment to enable precocious germination as per Munday et al. [10] and Ribalta et al. [11].

### Hydroponic experiments

A preliminary hydroponic experiment was conducted to establish a B concentration and duration of exposure that provided optimal discrimination among seven field pea genotypes (Additional file 1: Table S1) grown from mature seed. Seeds were nicked with a scalpel and imbibed for 24 h on DI water-moistened filter paper in petri dishes. For each of four B treatments, 10 seeds of each genotype were sown into 20 mm diameter peat plugs in ‘Preforma’ trays (J50000227, Garden City Plastics, Perth, Australia) floated in 40 L plastic crates containing 20 L of tap water, aerated using porous aquarium tubing. The location of genotypes were randomised within trays. The solution volume was maintained by adding tap water to replace evapotranspiration every two to three days. Plants were grown in a controlled environment room with 24/20 °C (day/night) temperature and a 20 h far-red enriched photoperiod supplied by LED lights (4:3 ratio of model 108D18-V12 tubes from S-Tech Lighting, Australia and AP67 L series tubes from Valoya, Helsinki, Finland; total intensity = 380 µmol m^−2^ s^−1^) (Additional file 2: Figure S1).

Seven days after imbibition, boric acid (H_3_BO_3_) dissolved in water was added to three treatments to bring the hydroponic solution to 5, 15 or 25 mg L^−1^ B. The final treatment was maintained as a nil B control. Leaf symptom ratings were recorded after 6 days of B exposure according to a 15-step scoring metric adapted from that used by Bagheri et al. [17] and Hobson et al. [23] (Additional file 3: Table S2). Symptom scores were analysed using ‘LSD.test’ (agricolae package) in RStudio (Version 0.99.484, RStudio, Inc.) with individual plants treated as replicates. A concentration of 15 mg L^−1^ B provided greatest discrimination among genotypes and was adopted for further hydroponic experiments.

Two subsequent hydroponic experiments (hereafter, ‘hydroponic experiment 1’ and ‘hydroponic experiment 2’) with a similar set of genotypes were conducted using the method described above to generate comparative rankings of B tolerance, assess the repeatability of the system and to test the effect of seed maturity on B symptoms (Additional file 1: Table S1). Six imbibed seeds for each genotype * maturity combination were imbibed and sown as previously described into 30 mm diameter peat plugs in ‘Preforma’ trays (J50001750, Garden City Plastics, Perth, Australia). Genotype * maturity combinations were randomised across two trays in separate 30 L storage crates containing 15 L of tap water. Plants were grown in identical culture conditions to those described above with one exception: the 15 mg L^−1^ B treatment was added 11 days after imbibition to provide seedlings more time to establish. Symptom scores were recorded and analysed as previously described. Due to insufficient germination in three genotype * maturity combinations in hydroponic experiment 2 (immature PBA Oura, immature Sturt and mature OZP1202) another small experiment was conducted with these combinations and their reciprocal maturity pairs. The results from missing combinations in hydroponic experiment 2 were filled using the results from the third experiment after an ‘LSD.test’ in RStudio confirmed that all three reciprocal maturity pairs in the third experiment were not significantly different (P > 0.05) from hydroponic experiment 2. A duplicate set of replicates were grown with no B added in all hydroponic experiments to act as a nil B control (B0).

The effect of the hydroponic B tolerance screen on time to floral initiation was assessed by transplanting into pots a subset of plants (Additional file 1: Table S1) that had undergone screening in hydroponic experiment 1 and comparing flowering behaviour to results generated during the development of the aSSD platform. Plants were transplanted the day after screening (18 days after imbibition) into 90 mm, free draining ‘Olive pots’ (P90OPX, Garden City Plastics, Australia) and grown as per Ribalta et al. [11] in the same environment used for the hydroponic experiments. Days to flowering (DTF) was recorded. For three genotypes (Additional file 1: Table S1), plants originating from both mature and immature seed were transplanted to soil and the effect of seed maturity on DTF was tested using one-way ANOVA in RStudio for each genotype. Other results for DTF of screened and transplanted plants were compared directly to DTF records obtained from plants grown as per Ribalta et al. [11] during the development and testing of the aSSD system.

### Pot experiments

For soil based experiments, mature seeds were imbibed for 24 h on filter paper moistened with DI water and sown in a glasshouse in 2 L sealed plastic pots filled with pasteurised potting mix (Richgro Garden Products Australia Pty Ltd) with B added as H_3_BO_3_. Five individuals were grown in separate pots for each treatment and genotype combination. The five replicates were blocked and each block completely randomised. The glasshouse was temperature controlled at 24/20 °C day/night with natural light conditions (July/August 2015; 12 h photoperiod). Immediately after sowing, pots were watered to 100% field capacity (fc) and allowed to dry down to 80% fc, after which pots were watered to 80% fc by weight every second or third day. Pots did not dry below 50% fc during the experiments.

A preliminary pot trial was conducted to identify a level of applied B that would enable clear discrimination between tolerant and susceptible genotypes. Two putatively tolerant (OZP1202 and PS3715) and one putatively susceptible (PBA Percy) field pea genotypes were subjected to four levels of applied B (20, 40, 60 or 80 mg B kg dry soil^−1^ applied as H_3_BO_3_) and compared to a control treatment (no applied B). Shoot and root dry weights were used to assess B sensitivity after 37 days’ growth. Boron application at 80 mg kg dry soil^−1^ provided the best discrimination among genotypes based on ‘LSD.test’ in RStudio and was selected for future pot based experiments.

A second pot-based experiment was conducted to examine the response of a larger selection of germplasm to B (Additional file 1: Table S1). Two treatments were used: addition of 80 mg B kg dry soil^−1^ (B80) and a control with no added B (B0). Six weeks after sowing, B toxicity symptoms in leaves were scored using the method outlined above (Additional file 3: Table S2) and roots and shoots were harvested for biomass measures. Plants were cut at the hypocotyl and roots and shoots rinsed with DI water before drying at 70 °C for one week to determine dry weight. The measures of biomass components of control (B0) plants were converted into a pooled mean for each genotype. A measure of the percentage depression of shoot and root growth due to the B treatment was then calculated for each B80 individual as the difference between the individual’s B80 biomass and the pooled mean of B0 plants, expressed as a percentage of B0 plants. Symptom scores and the depression of root and shoot growth of B treated plants were analysed using ‘LSD.test’ in RStudio to generate significance groupings (P = 0.05) among the genotypes.

To determine the effect of the B80 treatment on flowering time and seed yield, a pot-based experiment was conducted using two putatively tolerant (PBA Coogee and PS3715) and two putatively susceptible (PBA Percy and Sturt) genotypes. Experimental design and management was identical to the previous pot-based experiment, apart from the reduced subset of genotypes and extended growth to maturity. DTF was recorded on an individual plant basis, and watering ceased after a further 30 days. Dry seed pods were harvested and the number and weight of seeds produced by each plant recorded. Flowering time results were analysed for each genotype using one-way ANOVA in RStudio to test the effect of B on DTF. Seed weight and seed number were standardised as a percent of the control treatment by dividing the B80 individual measurements by the pooled average of control (B0) plants in the relevant genotype and multiplying by 100. These standardised data were then analysed using the ‘LSD.test’ in RStudio to create significance groups (P = 0.05) among the genotypes.

### Analysis of correlations among experiments

The strength and significance of correlations among the three B tolerance metrics measured in the pot experiments, the leaf symptom scores in hydroponic experiments and DEDJTR B tolerance rankings (Additional file 4: Table S3) were calculated using Pearson correlation coefficients in RStudio (‘Rcorr’ method in the ‘Hmisc’ package). DEDJTR data were assigned numerical ranks for the analysis viz. tolerant (1), moderately tolerant (2), moderately susceptible (3) and susceptible (4).

## Results

### Hydroponic experiments

The 15 mg L^−1^ B treatment provided the best discrimination among genotypes in the preliminary hydroponic experiment (5 significance levels and an overall range of 3.5 in scores, Table 1) and was selected for further hydroponic experiments. The most tolerant genotypes were PS3715 and OZP1202 and both were significantly more tolerant than Kaspa, PBA Oura, PBA Percy and Sturt, based on leaf symptoms.

**Table 1:** Boron (B) toxicity symptom scores (including the range of scores) of seven field pea genotypes subject to four B concentrations in a preliminary hydroponic experiment

Genotype	B0		B05		B15		B25	
	Score	Sig.	Score	Sig.	Score	Sig.	Score	Sig.
PBA Coogee	0	a	0	a	1.3	bc	2.6	b
Kaspa	0	a	0	a	2.2	cd	3.5	c
PBA Oura	0.1	ab	0.3	a	1.4	c	1.75	ab
OZP1202	0	a	0.2	a	0.4	ab	–	–
PBA Percy	0	a	1.6	b	3.6	e	4.7	d
PS3715	0.1	ab	0.1	a	0.1	a	1.7	a
Sturt	0.2	b	1.7	b	2.4	d	3.5	c
Range	0.2		1.7		3.5		3	

B0, B5, B15 and B25 refer to 0, 5, 15 and 25 mg L^−1^ B, respectively. Letters in ‘Sig.’ columns refer to significance groups among genotypes within each B treatment

**Fig. 1:**
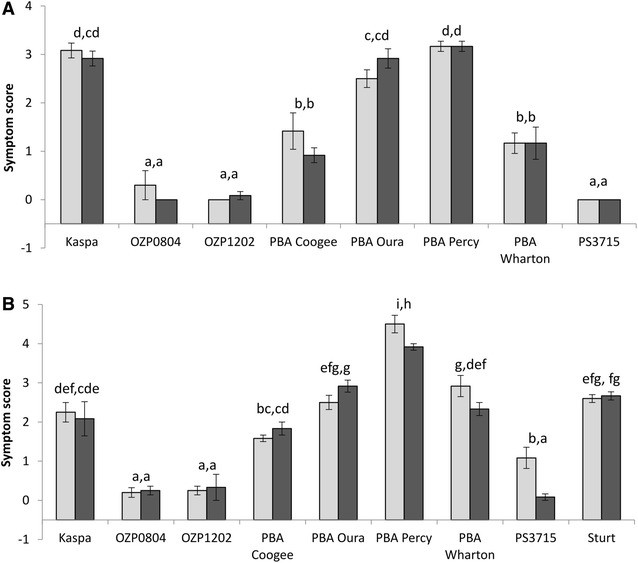
Boron toxicity symptom scores of field peas in hydroponic experiments 1 (**A**) and 2 (**B**) grown from immature (*light grey*) and mature (*dark grey*) seed. *Error bars* represent ± 1 standard error. *Letters* at top of bars indicate significance (P = 0.05) groupings of means (within each experiment) according to ‘LSD.test’ in RStudio. NB. X-axis crosses at Y = −0.5 to better illustrate genotypes with low average scores

Hydroponic experiment 1 provided three clear discrimination levels among genotypes based on leaf symptom scores (Fig. 1A; Additional file 5: Figure S2). PBA Percy, Kaspa and PBA Oura were the most susceptible genotypes, PBA Wharton and PBA Coogee were intermediate, and PS3715, OZP1202 and OZP0804 were the most tolerant. There was no significant effect of seed maturity on the B leaf toxicity symptom scores in any genotype.

Leaf symptoms in hydroponic experiment 2 (Fig. 1B) also provided significant discrimination among genotypes. Although the levels of discrimination were not as discrete as in hydroponic experiment 1, the main findings were consistent; PS3715, OZP1202 and OZP0804 were the most tolerant and Kaspa, PBA Oura and PBA Percy were among the least tolerant. Sturt, included in hydroponic experiment 2 but not hydroponic experiment 1, was also among the least tolerant genotypes. There were three cases where seed maturity had a significant effect on symptom scores in hydroponic experiment 2: PS3715, PBA Wharton and PBA Percy; although the magnitude of the differences was small relative to the differences among genotypes.

### Time to flowering in screened plants

The hydroponic B screening protocol reported here had no or minimal effect on time to floral initiation, compared to generations grown without a B stress in the aSSD system (Table 2). In all but one (PS3715) of the seven genotypes tested, DTF in screened plants was equivalent or lower than for aSSD generations without screening. Seed maturity had an effect on DTF in screened plants in PBA Percy (P < 0.002), with plants grown from immature PGS taking 5 days longer to flower (Table 2), while for PBA Coogee and PBA Oura, there was no effect of seed maturity on DTF (P ≥ 0.07).

**Table 2:** Days to flowering (DTF) for plants subject to hydroponic B screening then transplanted to pots (screened transplants), grown from immature precociously germinated seed (PGS) or mature seed, compared to plants grown in pots in an accelerated single seed descent (aSSD) system

Genotype	Screened transplants			aSSD^a^ DTF
	Immature PGS DTF (±SD)	Mature seed DTF (±SD)	Sig. (P value)	
PBA Coogee	43 (3.6)	41 (1.8)	0.42	43
Kaspa	–	37 (1.7)		41
PBA Oura	35 (3.8)	31 (0.8)	0.07	36
OZP0804	–	43 (2.6)		44
OZP1202	42 (2.2)	–		43
PBA Percy	33 (1.5)	28 (1.3)	0.002	35
PS3715	44 (2.9)	–		38

‘Sig.’ column shows *P* value of one-way ANOVA testing seed maturity effect on DTF

^a^‘aSSD’ flowering data taken from plants grown in pots and soil under aSSD conditions that minimise DTF as reported by Ribalta et al. [11], with no screening taking place

### Pot experiment

**Fig. 2:**
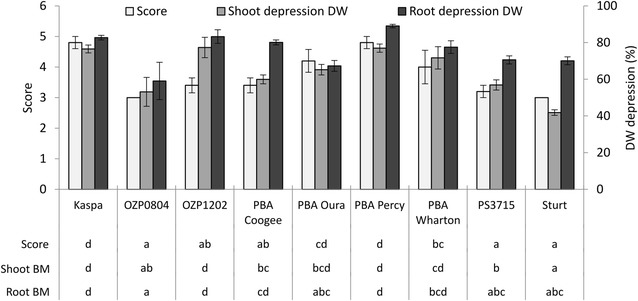
Mean leaf symptom scores (left hand y-axis), shoot (Shoot BM) and root (Root BM) dry weight depression (right hand y-axis) for nine field pea genotypes grown in pots with 80 mg kg^−1^ B amended soil. *Error bars* represent ± 1 standard error. *Letters* below genotype names indicate significance groupings among genotypes within each variable (labels on *left of lines*) based on ‘LSD.test’ in RStudio at P = 0.05

Figure 2 presents leaf symptom scores, shoot dry weight depression and root dry weight depression for the nine genotypes tested when grown in pots with 80 mg kg^−1^ B amended soil. There were significant differences among genotypes in all three metrics and on the whole, there was good consensus among the metrics. OZP0804, Sturt and PS3715 were significantly lower (more tolerant) in all three metrics, compared to Kaspa and PBA Percy. In leaf symptom scores, PBA Oura, Kaspa and PBA Percy displayed significantly more severe toxicity symptoms than OZP0804, Sturt, PS3715, PBA Coogee and OZP1202.

Leaf symptom scores from the pot experiment differed in scale and magnitude to those obtained in hydroponic experiments, with the lowest scores around 3 in the former and 0 in the latter. However, the discrimination of four significance levels among genotypes in the pot experiment was similar to that in hydroponic experiment 1. There were two obvious and specific inconsistencies in comparisons among the metrics in the pot experiment and the two hydroponic experiments. First, although Sturt appeared consistently tolerant in the pot experiment based on leaf symptom scores, biomass metrics and seed production (Fig. 2), this genotype had poor leaf symptom scores in both the preliminary hydroponic experiment (Table 1) and in hydroponic experiment 2 (Fig. 1B). Second, OZP1202 ranked poorly in biomass metrics in the pot experiment, but was consistently among the most tolerant genotypes based on leaf symptom scores in pots and all three experiments.

In plants grown to full maturity in pots, time to floral initiation was delayed by the B80 treatment in two (PBA Percy and Sturt) of the four genotypes tested (Table 3). Both seed count and seed weight were impacted by the B80 treatment in all four genotypes, with reductions around 70% or more. Seed production of PBA Coogee, PBA Percy and PS3715 were most heavily impacted by B, with no difference among these three genotypes. However, Sturt was able to maintain higher yield in the B80 treatment, with higher seed count than PBA Coogee and PBA Percy, and higher seed weight than the other three genotypes, proportional to the B0 control.

**Table 3:** Days to flower when grown in control (B0 DTF) and B amended (B80 DTF) potting mix, the significance of B effect on DTF (ANOVA P value), seed count and harvested seed weight for four field pea genotypes

Genotype	B0 DTF	B80 DTF	ANOVA P-value	Seed count (% control)	Sig.	Seed weight (% control)	Sig.
PBA Coogee	81.4	81.4	1.00	9.3	a	7.4	a
PBA Percy	65.6	74.2	0.01	4.6	a	4.4	a
PS3715	77.4	78.4	0.74	12.8	ab	9.3	a
Sturt	68	77.8	0.02	27.3	b	31.1	b

Seed count and weight are B80 results as a percent of control. Letters in ‘Sig.’ columns indicate significance groups among means within preceding column based on ‘LSD.test’ in RStudio (P = 0.05)

### Correlations between hydroponic scores and other results

The objective of this series of experiments was to validate B tolerance results based on a rapid hydroponic screening for leaf symptoms against a pot experiment and published rankings for the same genotypes, where available. All correlations among the tested B tolerance metrics were positive (Table 4). Leaf symptom scores from all three experiments and in both seed maturity levels were highly correlated (r > 0.7, P ≤ 0.05), except in one instance (Pot exp., Score vs. Hydro. exp. 2, Mat.) which was near significance (r = 0.66, P = 0.0514). Correlations among the metrics from the pot experiment were all near significance (root biomass vs. score: r = 0.62, P ≤ 0.1) or better (r ≥ 0.74, P ≤ 0.05). Biomass metrics from the pot experiment were not significantly correlated (P > 0.1) with metrics from hydroponic experiments or the DEDJTR data. All leaf symptom scores among the two hydroponic experiments were highly correlated (P < 0.05). DEDJTR data for B tolerance and symptom scores from both seed maturity levels in hydroponic experiment 1 were highly correlated (P ≤ 0.05), but DEDJTR data and hydroponic experiment 2 were less strongly correlated.

**Table 4:** Pearson correlation coefficients among B tolerance metrics measured in a pot experiment (Pot exp.) and two hydroponic experiments (Hydro. exp. 1 & 2) and B tolerance rankings from DEDJTR data and breeders’ advice, as described in the methods

	Hydro. exp. 1		Hydro. exp. 2		Pot exp.		
	Imm. PGS	Mat.	Imm. PGS	Mat.	Score	Shoot BM	Root BM
Hydro. exp. 1							
Imm. PGS	–						
Mat.	0.98***	–					
Hydro. exp. 2							
Imm. PGS	0.81*	0.82*	–				
Mat.	0.89**	0.90**	0.94***	–			
Pot exp.							
Score	0.93***	0.94***	0.70*	0.66^+^	–		
Shoot BM	0.50	0.54	0.25	0.20	0.76*	–	
Root BM	0.44	0.41	0.47	0.40	0.62^+^	0.74*	–
DEDJTR data	0.87*	0.84*	0.68^+^	0.65	0.50	0.21	0.51

Pot exp. metrics include leaf symptom scores (Score) and shoot and root biomass (BM) reduction relative to a control treatment. Hydroponic metrics were leaf symptom scores on plants grown from immature precociously germinated seed (Imm. PGS) or mature (Mat.) seed. DEDJTR data were converted to numerical categories as described in the methods. Symbols after correlation coefficients indicate significance levels (^+^ P ≤ 0.1; * P ≤ 0.05; ** P ≤ 0.01; *** P ≤ 0.001)

## Discussion

We present a protocol designed to enable high-throughput, hydroponic based, controlled environment screening for B toxicity leaf symptoms in field pea. We demonstrate that the use of precociously germinated seed (PGS) harvested 18 days after anthesis (DAA) yields results consistent with mature seed. The proposed screening protocol fulfils the requirements of integration within the accelerated single seed descent (aSSD) system proposed by Ribalta et al. [11]. In addition, leaf toxicity ratings across genotypes correlated well with B tolerance ratings from pot trials and published rankings [22]. We, therefore, accept our original hypotheses.

This is the first report of abiotic screening in pulses undertaken in environmentally regulated conditions with all photoperiod requirements met using far-red enriched artificial light sources. Boron screening under our controlled conditions represents a major timesaving compared to previously reported soil systems in field pea that took 28 days [24]. Plants could be scored within 17 days of seed imbibition and then transplanted to soil without substantial delay in days to floral initiation, compared with unscreened plants in a typical aSSD generation. The aSSD system makes use of controlled environments to tailor photoperiod, temperature and light quality to greatly reduce generation time and enable consistent year-round growth [9]. The use of controlled conditions for B tolerance screening in field peas provides similar benefits, resulting in a rapid, repeatable and consistent year-round screening platform.

The second major point of difference between our protocol and previously published screens is the use of immature PGS as the starting material. We found minimal impact on B tolerance rankings (based on leaf symptoms) when using seed harvested at 18 DAA and subjected to precocious germination treatment, compared to mature seed. Over 80% of the direct comparisons between immature and mature seed showed no significant difference in response of leaf symptoms to B exposure. In the few cases where there was a difference, the magnitude was small, relative to differences among genotypes. Correlations between rankings of mature and immature seed within both hydroponic experiments were above 0.81 and significant at P ≤ 0.05. Importantly, there was minimal delay in time to floral initiation in screened then transplanted seedlings grown from immature seed, compared to those grown from mature seed. We have demonstrated that screening immature seed for leaf toxicity symptoms provides a reliable discrimination for B tolerance without the delays associated with waiting for full seed maturity or with a flowering time penalty in the screened generation. Naturally, mature seed can also be used in the screening system if preferred.

Leaf symptom scores are routinely used in B tolerance screening in pulses [13, 16–18, 23–25]. We have validated our hydroponic screening methodology by comparison with leaf symptoms and biomass results in soil-grown plants. We have confirmed that leaf symptom results from hydroponically grown plants align well with published categories for B tolerance from DEDJTR data and our results from pot experiments. The leaf symptom scores from pot- and hydroponic-grown plants showed strong correlations for B tolerance and consistently identified the most tolerant genotypes. There were some specific inconsistencies in the performance of genotypes according to results from different metrics or experiments. For example, Sturt and OZP1202 showed differing responses depending on the metric and growth system used for screening (Table 1; Figs. 1, 2). It is possible that the tolerance expressed by Sturt in pot-grown plants is related to plant developmental stage, since seedlings of Sturt demonstrated poor tolerance in the hydroponic protocol. In the case of OZP1202, the reduced expression of B toxicity symptoms in leaves is apparently not linked with an ability to maintain growth under B stress, since biomass results in the pot experiment indicated that this genotype was among the most severely affected. These inconsistencies reinforce that B tolerance in field peas is not a simple mechanism and not fully understood [13]. Different mechanisms are likely to play a larger role under different conditions (soil vs. solution culture) and at different stages of plant development. Perhaps most importantly, leaf symptom scores from the pot experiment and the two hydroponic experiments consistently identified OZP0804, OZP1202 and PS3715 as being among the most tolerant genotypes tested. We have developed this screen to complement the aSSD process and anticipate its primary use will be as a rapid screening method to identify the extreme levels of tolerance within a population. The various limitations of screening seedlings for abiotic stresses, along with the advantages of high speed and throughput, must be fully considered when using a rapid, compact protocol such as that reported here.

Advances have been made to identify molecular markers for selection of B tolerance in a number of species [21, 26–30]. Tolerance to B is controlled by single genes in the model legume *Medicago truncatula* [21] and *Lens culinaris* [31], however, the molecular basis of B tolerance in field peas has not been firmly established. Bagheri et al. [24] hypothesised that two additive loci with incomplete dominance may be responsible for the B tolerance segregation ratios they observed. More recently, Sudheesh et al. [16] identified a major QTL for B tolerance in field pea and developed a putative marker for the region that was associated with both powdery mildew (PM) resistance and B tolerance. While validating this marker across a selection of germplasm and breeding lines, Javid et al. [3] confirmed a strong link between B tolerance and PM resistance (r = 0.959) but the marker incorrectly predicted B tolerance in 21% of cases. Concordant with screening results reported by Javid et al. [3], we found Kaspa and Sturt to be sensitive to B. However in that study, Sturt presented the B tolerant marker. This underlines the importance of combining a rapid phenotypic assay for B with molecular data to help breeders form a complete picture of the likely performance of genotypes in the field.

We believe that small modifications to the proposed technique will enable wider application to other pulse species for which an aSSD system is available (9, 10) and potentially make it applicable to screen for the individual effects of abiotic stresses other than B, or allow studies of the interactions between B tolerance and stresses such as salinity [32]. The vast majority of research on salinity and B tolerance has been undertaken independent of one another [33]. However, high salinity and high levels of B often co-occur in agricultural environments [34]. When both stresses occur together, studies have shown that increased salinity may reduce or increase boron’s toxicity effect [32].

## Conclusions

The B screening technique proposed herein will allow screening of RILs within an aSSD or traditional breeding system, speeding the integration of B stress tolerance into elite, locally adapted cultivars. The application of the B screen to an aSSD RIL population has the potential to further improve the efficiency of the aSSD process, as susceptible material can be quickly identified and discarded, reducing the numbers of RILs taken through the system from a particular cross combination. The position of the screening generation in the overall aSSD process is not critical and can be dictated by the specifics of the parental cross and the abiotic stress in question. For instance, screening for B tolerance may be undertaken early in the aSSD generations without high risk of removing tolerant material, since genetic control is fairly simple [3, 16, 24] compared to quantitative traits such as salinity tolerance. The key benefit of this protocol lies in the recoverability of the lines post-screening, the capacity to rapidly screen year-round and the integration with the existing aSSD system, forming a platform for rapid genetic improvement in field pea for this important trait.

## Additional files

**Additional file 1: Table S1.** List of genotypes used in a preliminary and two subsequent hydroponic experiments (‘Prelim. hydro.’, ‘Exp. 1’ and ‘Exp. 2’), a measurement of days to flower in transplanted seedlings (DTF) and each of three pot based experiments (‘Prelim. soil’, ‘Full trial’ and ‘DTF & yield’). ‘M’ and ‘I’ indicate that the experiment used plants grown from mature or immature seed, respectively.

**Additional file 2: Figure S1.** Spectrum of light used in controlled environments. Light spectrum in controlled growth environment used for hydroponic experiments modified from Croser et al. [9].

**Additional file 3: Table S2.** B toxicity scoring system. Leaf B toxicity scoring system used in hydroponic and pot experiments adapted from Hobson et al. [23] and Bagheri et al. [24].

**Additional file 4: Table S3.** DEDJTR boron tolerance rankings. DEDJTR boron tolerance rankings used as a benchmark for validation of B tolerance results obtained through hydroponic experiments. ‘DEDJTR data’ are published B tolerance ratings from Department of Economic Development Jobs Transport and Resources [22] : T = tolerant; MT = moderately tolerant; MS = moderately susceptible; S = susceptible.

**Additional file 5: Figure S2.** Example of B toxicity symptom expression in field peas. Field pea varieties subject to hydroponic boron (B) tolerance screening protocol. **a** PBA Oura, mature seed, grown in hydroponics with nil B. **b** PBA Oura, mature seed, with 6 days exposure to 15 mg L^−1^ B showing foliar toxicity symptoms on lower leaf margins (indicated by arrows). **c** OZP1202, mature seed, with 6 days exposure to 15 mg L^−1^ B showing only minor chlorosis symptoms.
