# Supplementary material for: Discrimination of boron tolerance in Pisum sativum L. genotypes using a rapid, high-throughput hydroponic screen and precociously germinated seed grown under far-red enriched light
Source: Plant Methods. 2017 Aug 29;13:70. doi: 10.1186/s13007-017-0221-3 (PMC5575881; doi:10.1186/s13007-017-0221-3)
Supplement: Supplementary file 1 — Additional file 1: Table S1. List of genotypes used in a preliminary and two subsequent hydroponic experiments (‘Prelim. hydro.’, ‘Exp. 1’ and ‘Exp. 2’), a measurement of days to flower in transplanted seedlings (DTF) and each of three pot based experiments (‘Prelim. soil’, ‘Full trial’ and ‘DTF & yield’). ‘M’ and ‘I’ indicate that the experiment used plants grown from mature or immature seed, respectively. [file 13007_2017_221_MOESM1_ESM.docx]

Table S1. List of genotypes used in a preliminary and two subsequent hydroponic experiments (‘Prelim. hydro.’, ‘Exp. 1’ and ‘Exp. 2’), a measurement of days to flower in transplanted seedlings (DTF) and each of three pot based experiments (‘Prelim. soil’, ‘Full trial’ and ‘DTF & yield’). ‘M’ and ‘I’ indicate that the experiment used plants grown from mature or immature seed, respectively.

| Genotype | Hydroponic experiment | | | | Pot experiment | | |
| --- | --- | --- | --- | --- | --- | --- | --- |
|  | Prelim. hydro. | Exp. 1 | Exp. 2 | DTF | Prelim. soil | Full trial | DTF & yield |
| Kaspa | M | M & I | M & I | M |  | M |  |
| OZP0804 | M | M & I | M & I | M |  | M |  |
| OZP1202 |  | M & I | M^1^ & I | I | M | M |  |
| PBA Coogee | M | M & I | M & I | M & I |  | M | M |
| PBA Oura | M | M & I | M & I^1^ | M & I |  | M |  |
| PBA Percy | M | M & I | M & I | M & I | M | M | M |
| PBA Wharton | M | M & I | M & I |  |  | M |  |
| PS3715 | M | M & I | M & I | I | M | M | M |
| Sturt | M |  | M & I^1^ |  |  | M | M |

^1^ – Results from these genotype and maturity combinations were obtained in a third hydroponic experiment due to poor germination in the second hydroponic experiment.
