# Supplementary figures and images for: Discrimination of boron tolerance in Pisum sativum L. genotypes using a rapid, high-throughput hydroponic screen and precociously germinated seed grown under far-red enriched light
Source: Plant Methods. 2017 Aug 29;13:70. doi: 10.1186/s13007-017-0221-3 (PMC5575881; doi:10.1186/s13007-017-0221-3)

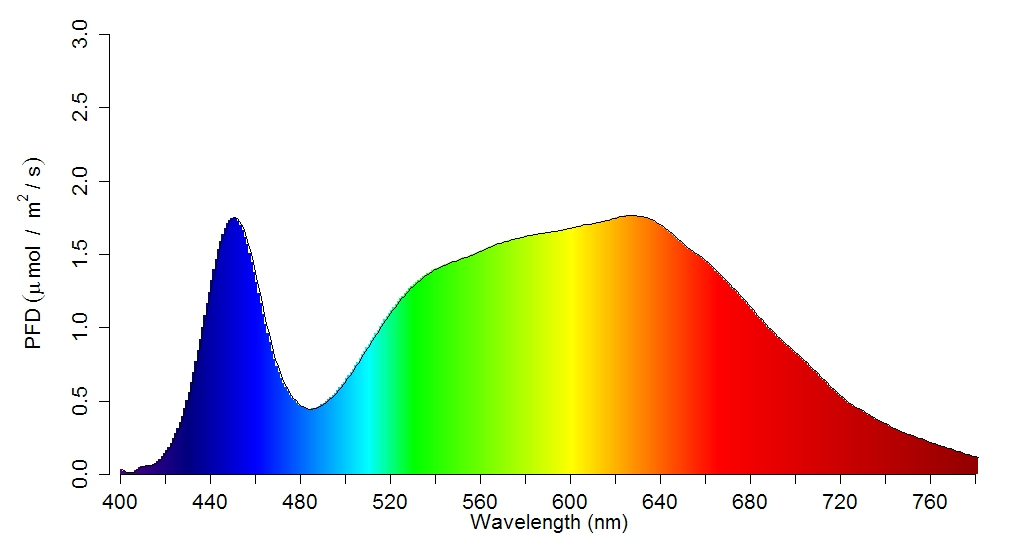

Supplement: Supplementary file 2 — Additional file 2: Figure S1. Spectrum of light used in controlled environments. Light spectrum in controlled growth environment used for hydroponic experiments modified from Croser et al. [9]. [file 13007_2017_221_MOESM2_ESM.jpeg]

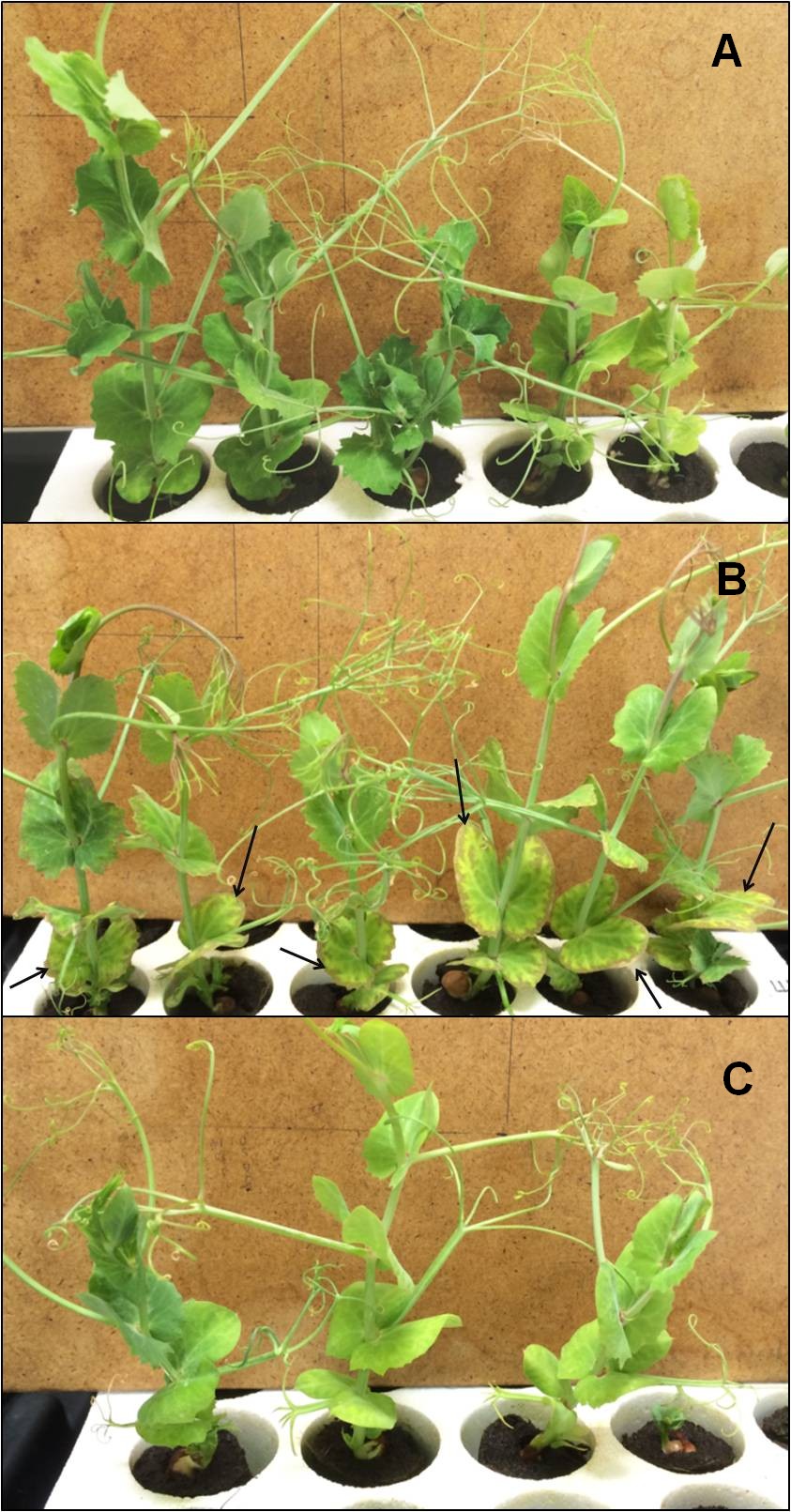

Supplement: Supplementary file 5 — Additional file 5: Figure S2. Example of B toxicity symptom expression in field peas. Field pea varieties subject to hydroponic boron (B) tolerance screening protocol. a PBA Oura, mature seed, grown in hydroponics with nil B. b PBA Oura, mature seed, with 6 days exposure to 15 mg L−1 B showing foliar toxicity symptoms on lower leaf margins (indicated by arrows). c OZP1202, mature seed, with 6 days exposure to 15 mg L−1 B showing only minor chlorosis symptoms. [file 13007_2017_221_MOESM5_ESM.jpg]
