# Supplementary material for: Discrimination of boron tolerance in Pisum sativum L. genotypes using a rapid, high-throughput hydroponic screen and precociously germinated seed grown under far-red enriched light
Source: Plant Methods. 2017 Aug 29;13:70. doi: 10.1186/s13007-017-0221-3 (PMC5575881; doi:10.1186/s13007-017-0221-3)
Supplement: Supplementary file 3 — Additional file 3: Table S2. B toxicity scoring system. Leaf B toxicity scoring system used in hydroponic and pot experiments adapted from Hobson et al. [23] and Bagheri et al. [24]. [file 13007_2017_221_MOESM3_ESM.docx]

Table S2. Leaf B toxicity scoring system used in hydroponic and pot experiments adapted from Hobson et al. [23] and Bagheri et al. [24].

| Score | Symptom stage |
| --- | --- |
| 0 | No apparent symptoms |
| 0.5 | Chlorosis on tips of worst leaf, no marginal necrosis |
| 1.0 | Tip necrosis on the worst leaf |
| 1.5 | As 1.0, plus chlorosis on tips of a second set of leaves |
| 2.0 | Tip necrosis on a second set of leaves and leaf necrosis on less than or equal to 25% of the worst leaf |
| 2.5 | As 2.0, plus chlorosis on tips of a third set of leaves |
| 3.0 | Tip necrosis on a third set of leaves and leaf necrosis on 26 to 50% of the worst leaf |
| 3.5 | As 3.0, plus tip necrosis on a fourth set of leaves |
| 4.0 | As 3.5, plus leaf necrosis on 51 to 75% of the worst leaf |
| 4.5 | As 4.0, plus tip necrosis on a fifth set of leaves |
| 5.0 | As 4.5, plus leaf necrosis on greater than 76% of the worst leaf or leaves beginning to drop |
| 5.5 | All leaves with marginal necrosis except youngest leaves |
| 6.0 | Plant wilted |
| 7.0 | Only stem green |
| 8.0 | Plant dead |

NB. Multiple leaflets were considered as one leaf in genotypes with pinnate leaves and a pair of stipules was counted as a single leaf in leafless genotypes.
