# Supplementary material for: Discrimination of boron tolerance in Pisum sativum L. genotypes using a rapid, high-throughput hydroponic screen and precociously germinated seed grown under far-red enriched light
Source: Plant Methods. 2017 Aug 29;13:70. doi: 10.1186/s13007-017-0221-3 (PMC5575881; doi:10.1186/s13007-017-0221-3)
Supplement: Supplementary file 4 — Additional file 4: Table S3. DEDJTR boron tolerance rankings. DEDJTR boron tolerance rankings used as a benchmark for validation of B tolerance results obtained through hydroponic experiments. ‘DEDJTR data’ are published B tolerance ratings from Department of Economic Development Jobs Transport and Resources [22] : T = tolerant; MT = moderately tolerant; MS = moderately susceptible; S = susceptible. [file 13007_2017_221_MOESM4_ESM.docx]

Table S3. DEDJTR boron tolerance rankings used as a benchmark for validation of B tolerance results obtained through hydroponic experiments. ‘DEDJTR data’ are published B tolerance ratings from Department of Economic Development Jobs Transport and Resources [22] : T = tolerant; MT = moderately tolerant; MS = moderately susceptible; S = susceptible.

| Genotype | DEDJTR data |
| --- | --- |
| Kaspa | S |
| OZP0804 | T |
| PBA Coogee | T |
| PBA Oura | MS |
| PBA Percy | S |
| PBA Wharton | MT |
| Sturt | S |
